# Societal Preferences, Values and Priorities for Genomic Testing for Atrial Fibrillation: Evidence from Two Discrete Choice Experiments

**DOI:** 10.1007/s40271-026-00801-w

**Published:** 2026-02-13

**Authors:** Cun Liu, Stephanie Best, Diane Fatkin, Ilias Goranitis

**Affiliations:** 1 Economics of Genomics and Precision Medicine Unit, Centre for Health Policy, Melbourne School of Population and Global Health, University of Melbourne, Melbourne, VIC Australia; 2 Melbourne School of Health Sciences, University of Melbourne, Melbourne, VIC Australia; 3 Victor Chang Cardiac Research Institute, Darlinghurst, NSW Australia; 4 School of Clinical Medicine, Faculty of Medicine and Health, UNSW Sydney, Kensington, NSW Australia; 5 Cardiology Department, St Vincent’s Hospital, Darlinghurst, NSW Australia; 6 Australian Genomics, Murdoch Children’s Research Institute, Melbourne, VIC Australia

## Abstract

**Background and Objective:**

Atrial fibrillation is the most common sustained cardiac arrhythmia and is associated with an increased risk of stroke, heart failure and death. Genetic factors can contribute to atrial fibrillation pathogenesis and have the potential for atrial fibrillation risk stratification and clinical management. Using stated preference methods, this paper provides the first empirical evidence on consumer preferences, values and priorities for genomic testing in atrial fibrillation.

**Methods:**

Two discrete choice experiment surveys were designed and administered to the Australian public. Participants were asked to imagine situations where they either developed atrial fibrillation symptoms (symptomatic patients survey, *n* = 503) or had close family members diagnosed with atrial fibrillation (at-risk relatives survey, *n* = 506). Each participant was given 12 hypothetical scenarios involving five key aspects of genomic testing. Choice data were analysed using panel error component mixed logit and latent class models.

**Results:**

The two most valued benefits were clinical implications for monitoring personal cardiac complications other than atrial fibrillation and health implications for other family members. Cost was the main driver of preferences for testing. The value of genomic testing was estimated at $2900 for symptomatic patients and approximately 10% less for at-risk relatives. Uptake was predicted at around 82% for both groups if the test was publicly funded. People of lower financial or educational status and people without private health insurance were less likely to take-up genomic testing.

**Conclusions:**

Genomic testing for atrial fibrillation has perceived value for symptomatic patients and at-risk relatives. Tailored educational programmes and targeted support are essential to improve access for socioeconomically disadvantaged groups.

**Supplementary Information:**

The online version contains supplementary material available at 10.1007/s40271-026-00801-w.

## Key Points for Decision Makers

**Table:** 

Participants showed preference for the health and non-health outcomes of genomics for atrial fibrillation.
Clinical implications for monitoring personal cardiac complications other than atrial fibrillation and health implications for other family members were the two most valued outcomes of genomics. Cost was the most significant determinant of people’s decision to take-up genomic testing.
The outcomes of genomics are highly valued; AU$2900 for genomic testing in symptomatic patients and 10% less (AU$2600) for at-risk relatives. A publicly funded genomic testing uptake was predicted at 82%.

## Introduction

Atrial fibrillation (AF) is the most prevalent cardiac arrhythmia, and is characterised by rapid and irregular atrial activation [1]. Atrial fibrillation is associated with an increased risk of stroke, heart failure and death, contributing significantly to cardiovascular morbidity and mortality [2, 3]. Advances in genome sequencing have greatly enhanced our understanding of the genetic factors involved in the pathogenesis of AF [4, 5]. With increasing accessibility and affordability of sequencing technologies, there are emerging opportunities for genetics information to be incorporated into AF risk stratification and clinical management decisions [6, 7]. To effectively integrate genomic testing into mainstream clinical care for AF, it is essential to understand consumer preferences for the diagnostic, clinical and personal outcomes generated. This includes factors such as knowledge about the condition and its progression, and the health implications for family members [8]. The value of the diagnostic, clinical and non-clinical components of genomic testing is commonly referred to as genomic utility [9]. However, there are currently no data available on the genomic utility associated with the risks and benefits of genomic testing in AF.

A discrete choice experiment (DCE) is an established survey-based preference elicitation method to quantify the value of healthcare-related components by analysing how choices are made between different healthcare options using econometric methods [10, 11]. Discrete choice experiments have been increasingly employed to understand preferences, values and priorities for genomic testing across a variety of clinical contexts [12–17]. However, the evidence base for genomic testing in cardiovascular diseases remains limited [18–20], and no studies have examined preferences for genomic testing in the context of AF.

In a DCE, healthcare interventions can be described by a combination of characteristics (i.e. attributes) and associated measures to describe each attribute (i.e. levels). For example, the cost of testing could be an attribute with levels such as AU$500, AU$1000 or AU$2000. Survey respondents are asked to choose their preferred option from two or more alternatives consisting of different combinations of attributes-levels. Discrete choice experiments rely on an economic theory of human behaviours, which assumes that individuals will choose the option that maximises their underlying (latent) utility. Monetary measures of utility can be estimated to quantify how much risk respondents are willing to accept in order to receive particular benefits of genomic testing for AF, and to predict the uptake of testing.

Using hypothetical contexts where participants were asked to assume they developed symptoms (symptomatic patients survey) or had close family members diagnosed with AF (at-risk relatives survey), we conducted two DCEs to: (1) assess the relative importance of the diagnostic, prognostic, clinical and family attributes in determining consumer preferences for genomic testing in AF; (2) determine society’s value and uptake of genomic testing; and (3) examine how personal sociodemographic and attitudinal characteristics influence preferences for genomic testing. Our study provides the first empirical evidence on the economic value of the clinical and non-clinical outcomes of genomic testing for AF. Our findings can be used to inform cost-benefit analysis and policy decisions on the translation of genomic testing for AF within the healthcare system.

## Methods

### Study Design and Participants

Two DCE surveys were developed in the hypothetical context of having AF symptoms (symptomatic patients) and having close family members with AF (at-risk relatives) to elicit preferences, values and priorities for genomic testing in AF in accordance with best research practices [21] and reporting recommendations [22]. In DCEs, respondents are presented with a series of hypothetical choice scenarios (choice tasks), which are specified by two or more alternatives involving different combinations of attributes and associated levels [23]. In each choice task, respondents are asked to choose their preferred alternative. Discrete choice methods assume that each respondent will make choices based on their derived utility from the alternatives, which can be expressed as a mathematical function of each attribute. The impact of change in the attribute levels on choice, and the resulting equivalent monetary values, willingness to pay (WTP), can then be quantified from an econometric analysis of responses across different choice tasks [24].

To generate attributes for the DCEs, a series of focus groups were used to determine factors that influence people’s preferences for (or against) genomic testing [25]. In summary, six deliberative focus groups were conducted with members of the Australian public, recruited through advertisement on the websites and social media accounts of associated institutes (University of Melbourne and Victor Chang Cardiac Research Institute). In total, 14 individuals participated across the first four focus groups exploring the risks and benefits associated with genomic testing for AF, and 12 individuals participated across the final two groups (11 returning participants and one new participant) to refine and reframe the attributes identified in the earlier sessions. We developed a focus group protocol based on the existing literature and inputs from experts within the research team to guide and promote the discussion and inform the quantitative characteristics ranking. An iterative and co-productive approach was used to develop the selection and labelling of attributes with all focus group participants. Full details of the focus groups are provided elsewhere [26]. We then worked closely with the genetic experts to finalise the attributes and attribute levels to ensure clinical face validity. The final five attributes and levels are presented in Table 1.

**Table 1:** Attributes and attribute levels included in the discrete choice experiment

Attributes	Levels
1. Number of people who receive a genetic diagnosis	5 out of 100
	10 out of 100
	20 out of 100
	35 out of 100
2. Knowledge about future recurrence of AF and disease progression	Yes
	No
3. Consequences of the genetic diagnosis to you	Inform changes in AF treatment and management
	Inform ongoing monitoring to detect other heart complications that are related to the genetic diagnosis of AF
	Inform changes in treatment of co-morbidities and other risk factors that predisposed to AF
	Inform lifestyle changes to avoid triggers of AF
	No consequence
4. Consequences of the genetic diagnosis to your family	Identify the risk of developing AF and initiate baseline cardiac investigation
	No consequence
5. Cost of testing to you	AU$500
	AU$1500
	AU$3000
	AU$4500

*AF* atrial fibrillation

**Fig. 1:**
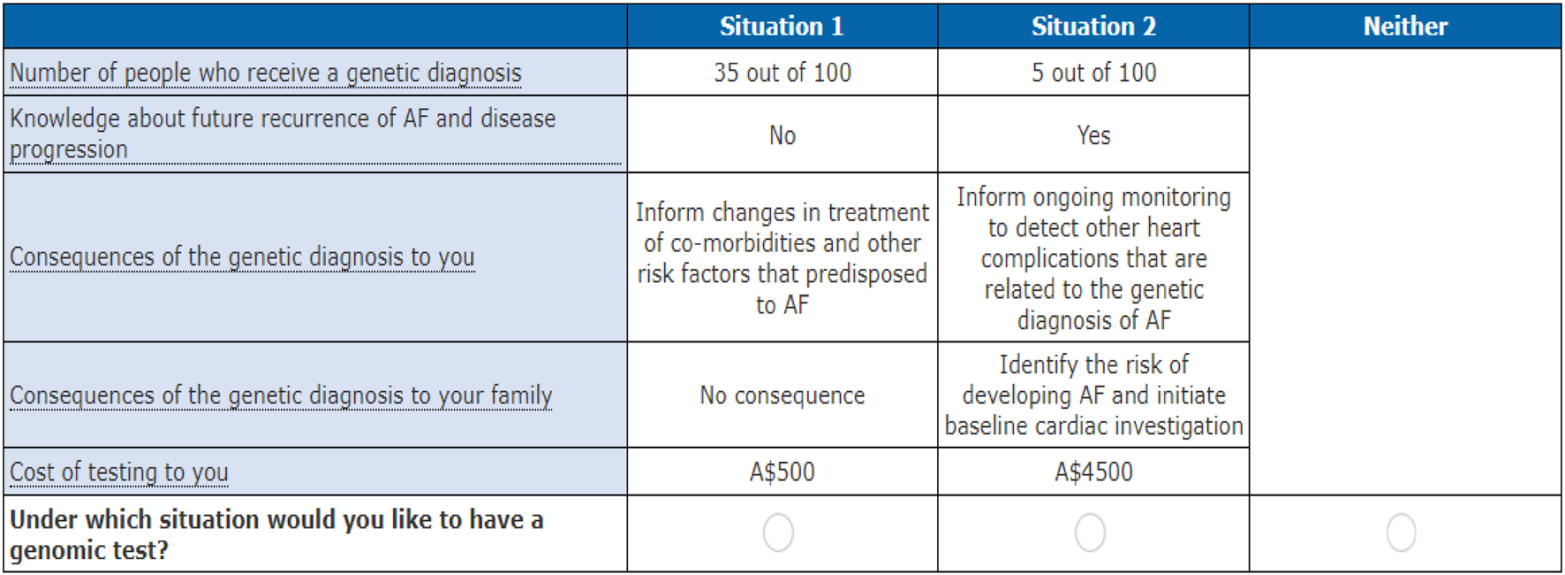
Choice task example. *AF* atrial fibrillation

A Bayesian D-efficient design was adopted to develop the choice tasks using Ngene (Choice Metrics [2018] Ngene 1.2 User Manual & Reference Guide, Australia). By assuming prior distributions of parameter values and optimising the design over the distribution, the Bayesian efficient design can account for uncertainties to ensure the robustness of design and reliability of estimated parameters. Published evidence and the expectations of the research team were used to inform the Bayesian priors for the pilot survey [27]. For each survey, there were 36 choice tasks split into three blocks to reduce respondent burden. The choice tasks were tailored to each survey to allow for different preferences across the symptomatic patients and at-risk relatives contexts^1^. We added a dominant choice task to test for internal validity. Thus, each participant had to complete 13 choice tasks. In each choice task, we asked participants to choose the situation under which they would like to have a genomic test. They could choose either one of the two genomic testing situations (Situation 1 or Situation 2) or the opt-out option (“Neither”), which represented the current standard of care where genomic testing is not readily available. Figure 1 shows an example of a choice task.

The DCE surveys included the following components: (1) demographics and questions on experiences with genomic tests and AF; (2) quality check question; (3) background information on how it is to live with AF, the impact AF has and the causes of AF; (4) detailed information on attributes and attribute levels; (5) an example choice task to help the respondent understand how to make trade-offs between different combinations of attributes and attribute levels in choice scenarios; (6) choice tasks. The differences between the two surveys were the background information and and choice tasks that are specifically developed to align with the objectives of each survey. The final version of the surveys was compiled in consultation with all members of the research team and focus group members. The two surveys surveys were piloted with 107 members of the Australian public (symptomatic, *n* = 55; at risk, *n*=52), recruited through an online panel firm (Pureprofile). Estimates from pilot data were used to assess the reliability of the Bayesian prior specifications. Minor edits were made on the wording of levels in the clinical consequence attribute based on the pilot result. The symptomatic patients survey is provided as an example in the Electronic Supplementary Material (ESM).

The symptomatic and at-risk surveys were administered to members of the Australian general public aged over 18 years, recruited through an online nationwide panel managed by Pureprofile. Both samples were stratified by age, sex, income, and geographic location to ensure representativeness of the Australian context. Participants were asked to imagine that they had developed AF symptoms (symptomatic patients survey) or that had a close family member diagnosed with AF (at-risk relatives survey). The S-efficiency measure was employed to determine the minimum sample size required to achieve statistically significant parameter estimates at the 5% level of significance [28]. In each of the symptomatic and at-risk surveys, participants were initially randomised into each of the three blocks. Within each block, we further applied the randomisation in the order of choice tasks, and in the order of the two genomic testing situations to account for left-to-right bias.

### Choice Data Analysis

A panel error component mixed logit (ECML) model was employed to estimate the marginal utility associated with each attribute using NLOGIT 6 (Econometric Software, Inc., Waverton, NSW, Australia). The panel ECML model captures the panel nature of the choice data and accounts for the correlation between the two genomic testing situations through the error component. The random parameters used in the model further incorporates unobserved heterogeneity of preferences among individuals by allowing estimated coefficients to vary around a specific distribution [21]. For the random parameter associated with the $kth$ attribute level (${\beta }_{k,i}={\beta }_{k}+{\sigma }_{k}{v}_{k,i})$, the mean (${\beta }_{k}$) and standard deviation (SD, ${\sigma }_{k}$) associated with the specified distribution ${v}_{k,i}$ were estimated from the ECML model. The mean (${\beta }_{k}$) represents the average marginal utility over the sampled population, while the SD (${\sigma }_{k}$) reflects the dispersion around the mean. A significant ${\sigma }_{k}$ indicates preference heterogeneity within the sampled population (i.e. different individuals have specific parameter estimates that may be different from the mean). For the cost of testing attribute, a constrained triangular distribution (${V}_{i}\sim T[-\mathrm{1,0}]$) was applied to ensure that participants have a negative preference for extra costs associated with genomic testing. A normal distribution was assumed for the other attributes (${V}_{i}\sim N[\mathrm{0,1}]$). We used 500 standard Halton draws to estimate the random parameters. Dummy coding was applied for the attributes “*Knowledge about future recurrence of AF and disease progression*”, “*Consequences of the genetic diagnosis to you*”, and “*Consequences of the genetic diagnosis to your family*”. “*Number of people who receive a genetic diagnosis*” and “*Cost of testing to you*” were continuously coded. The primary ECML model included the DCE attributes as main effects, and we conducted an additional analysis to control for sociodemographic attitudinal variables and experiences related to genetic and heart conditions.

The marginal value of each attribute, expressed as WTP, was estimated using a WTP version of the utility function in the ECML model, which provides a more reliable distribution for WTP [29]. To estimate the relative importance of each attribute, the importance scores were calculated by dividing the differences in estimated utility between the best and worst attribute level by the sum of the utility differences across attributes, using mean marginal utility estimates from the primary ECML model. Therefore, our relative importance values reflected both the importance of the attributes and the spread of levels. The overall monetary value of the benefits of testing, measured as incremental WTP for genomic testing relative to standard care of AF, were estimated under different clinical contexts [30]. The clinical scenarios were constructed based on current knowledge and evidence of diagnostic yield, clinical and familial consequences in consultation with the genetic specialist (DF) from the research team. The incremental WTP estimates were computed using the compensating variation formula [31, 32], and the delta method was employed to generate the 95% confidence intervals (CIs). We further validated the WTP values through population simulation, which accounted for estimated preferences heterogeneity, and robust regressions with S-estimator were used to minimise the effect of outliers on the simulated data [33]. Marginal and incremental WTP values are reported in Australian dollars ($AU). We also constructed testing uptake across the general population and within specific sociodemographic subgroups. Uptake was estimated as the predicted percentage of individuals choosing genomic testing and was graphed as a function of cost [30].

To further investigate participants’ preference heterogeneity, we estimated a latent class (LC) model and a fractional logit regression model. The LC model split individuals into a finite number of groups with distinct preferences for genomic testing in AF [34]. Individuals within each of these groups were assumed to have homogenous preference. The LC model was estimated using three classes based on the Bayesian information criterion and theoretical interpretability. Each respondent was assigned to the class with the highest probability. We then used a fractional logistic regression to investigate the association between individual characteristics and probability of class membership [35].

## Results

### Demographics

Out of 1017 individuals who completed the surveys, a total of 1009 respondents were included in our primary analysis for symptomatic patients (*N* = 503) and at-risk relatives (*N* = 506); eight participants who failed the quality check question were excluded^2^. The composition of the sociodemographic and attitudinal characteristics was not significantly different between the symptomatic and at-risk samples. The average age was 48 years (SD = 17), with around half of respondents being female (50% and 53%), having a university-level education (43% and 47%) and an annual household income above AU$100,000. The majority of participants resided in metropolitan areas (72%). Over 60% of respondents were married or in a de facto relationship (62% and 63%), parents (65% and 67%) and with private health insurance (63% and 69%). Approximately 20% of respondents reported having an experience with AF (21% and 20%), either directly (themselves) or indirectly (close family members or friends), while over 50% reported an experience of other heart conditions (53% and 57%). Over 35% of the sample indicated having personal experience or experience from close members/friends with a genetic condition (36% and 39%), and around 24% had had some form of genetic testing. Overall, study participants were relatively more educated compared with the national census summary. We used the census data to adjust the education sampling weights in our analysis so that they more accurately represented the Australian context. Additional information about the participants characteristics is provided in Table S1 of the ESM.

### Estimation of Mixed Logit Results, Willingness to Pay and Uptake

#### Preference for Genomic Testing

Table 2 shows the marginal utility of each attribute and levels from the panel ECML model. All estimated mean marginal utilities were significant at the 1% level of statistical significance. As expected, participants had a negative preference for genomic testing when the cost is higher. On average, participants in both the symptomatic and at-risk surveys preferred to take-up genomic testing when: (i) more patients with AF could receive a genetic diagnosis; (ii) knowledge about the future recurrence of AF and disease progression was able to be provided; (iii) there were positive consequences for patients with AF from the genetic diagnosis; and (iv) family members could learn about their risk for developing AF, which would prompt them to initiate baseline cardiac investigations. Overall, participants in the symptomatic survey showed a greater preference for those attributes compared with the at-risk survey. In the symptomatic survey, preferences were homogenous for the clinical consequences that informed the treatment of co-morbidities and other risk factors predisposing to AF, ongoing monitoring to detect other heart complications, and changes in AF treatment and management. This is evidenced by the non-statistically significant SD (*p* > 0.1) estimates for those attribute levels, indicating that marginal utility gains for those attribute levels did not vary significantly among participants in the symptomatic survey. In the at-risk survey, there were no statistically significant variations in participants’ preferences regarding how genetic diagnosis could inform changes in lifestyle and treatment for co-morbidities and other risk factors related to AF.

**Table 2:** Mixed logit regression with main attributes

Attributes		Symptomatic (*n* = 503)	*P*-value	At risk (*n* = 506)	*P*-value
Number of people who receive a genetic diagnosis	Mean	0.013 (0.007, 0.018)	< 0.00001	0.015 (0.009, 0.02)	< 0.00001
	SD	0.035 (0.028, 0.041)	< 0.00001	0.029 (0.023, 0.035)	< 0.00001
Knowledge about future recurrence of AF and disease progression (yes)	Mean	0.542 (0.436, 0.647)	< 0.00001	0.452 (0.358, 0.546)	< 0.00001
	SD	0.459 (0.325, 0.593)	< 0.00001	0.376 (0.217, 0.535)	< 0.00001
*Consequences of the genetic diagnosis to you*					
Inform lifestyle changes to avoid triggers of AF	Mean	0.641 (0.449, 0.834)	< 0.00001	0.515 (0.334, 0.696)	< 0.00001
	SD	0.776 (0.55, 1.002)	< 0.00001	0.041 (− 0.633, 0.715)	0.9043
Inform changes in treatment of co-morbidities and other risk factors that predisposed to AF	Mean	0.912 (0.728, 1.096)	< 0.00001	0.732 (0.578, 0.887)	< 0.00001
	SD	0.29 (− 0.072, 0.651)	0.116	0.118 (− 0.452, 0.687)	0.6853
Inform ongoing monitoring to detect other heart complications that are related to the genetic diagnosis of AF	Mean	1.09 (0.901, 1.278)	< 0.00001	0.92 (0.712, 1.127)	< 0.00001
	SD	0.1 (− 0.476, 0.677)	0.7334	0.373 (− 0.037, 0.783)	0.0748
Inform changes in AF treatment and management	Mean	0.737 (0.522, 0.952)	< 0.00001	0.746 (0.536, 0.956)	< 0.00001
	SD	0.262 (-0.213 to 0.737)	0.28	0.6 (0.345, 0.854)	< 0.00001
No consequence	Base				
*Consequences of the genetic diagnosis to your family*					
Identify the risk of developing AF and initiate baseline cardiac investigation	Mean	0.909 (0.77, 1.048)	< 0.00001	0.78 (0.64, 0.919)	< 0.00001
	SD	0.943 (0.813, 1.073)	< 0.00001	1.006 (0.875, 1.136)	< 0.00001
No consequence	Base				
Cost of testing to you (AU$1000s)	Mean	− 1.16 (− 1.23, − 1.08)	< 0.00001	− 1.24 (− 1.33 to − 1.15)	< 0.00001
	SD	1.16 (1.08, 1.23)	< 0.00001	1.24 (1.15, 1.33)	< 0.00001
Genomic testing constant	Mean	2.541 (2.026, 3.055)	< 0.00001	2.431 (1.932, 2.929)	< 0.00001
	SD	4.157 (3.568, 4.746)	< 0.00001	1.83 (1.174, 2.486)	< 0.00001
Log likelihood function	− 3913.455			− 4079.929	
McFadden pseudo R-squared	0.41			0.388	
Akaike information criterion	7862.9			8195.9	

Mixed logit regression analysis to assess the marginal utility with each attribute and preference heterogeneity among participants. All parameters are estimated as random parameters ${(\beta }_{k,i}={\beta }_{k}+{\sigma }_{k}{v}_{k,i})$. The mean (${\beta }_{k}$) and SD (${\sigma }_{k}$) associated with the specified distribution ${v}_{k,i}$ were estimated. The mean (${\beta }_{k}$) represents the average marginal utility over the sampled population, while the SD (${\sigma }_{k}$) reflects the dispersion around the mean. A significant SD (*P* < 0.1) indicates preference heterogeneity within the sampled population (i.e. different individuals have specific parameter estimates that may be different from the mean. For the cost of testing attribute, a constrained triangular distribution (${V}_{i}\sim T[-\mathrm{1,0}]$) was applied to ensure that participants have a negative preference for extra costs associated with genomic testing. A normal distribution was assumed for the other attributes (${V}_{i}\sim N[\mathrm{0,1}]$)

*AF* atrial fibrillation, *SD* standard deviation

We also estimated a panel ECML model additionally controlling for participants’ sociodemographic and attitudinal characteristics. The results further confirm the robustness of the findings discussed above (Table S2 of the ESM) and suggested that, for both the symptomatic and at-risk surveys, on average, being older and female were associated with a perceived lower utility of testing. Individuals who are married or in a de facto relationship and those with private health insurance had on average a higher utility for testing compared with those without a partner and private health insurance. Having children and attitudes towards health risks positively influenced utility for genomic testing, though this was not significant in the at-risk survey.

The relative importance of each attribute on a person’s decision to undergo genomic testing for AF was computed from estimated marginal utilities (Table 3). For both the symptomatic and at-risk samples, apart from the cost, the most important attribute was *“consequence of the genetic diagnosis to you”*, followed by *“consequence of the genetic diagnosis to your family”*, *“knowledge about future recurrence of AF and disease progression”* and *“number of people who receive a genetic diagnosis”*.

**Table 3:** Marginal WTP and importance score

Attributes	Symptomatic (*n* = 503)			At risk (*n* = 506)		
	Marginal WTP, AU$	*P*-value	Importance score, %	Marginal WTP, AU$	*P*-value	Importance score, %
Number of people who receive a genetic diagnosis	13 (6–19)	0.0001	5.0%	11 (5–17)	0.0002	5.8%
Knowledge about future recurrence of AF and disease progression (yes)	619 (482–755)	< 0.00001	7.2%	440 (328–551)	< 0.00001	6.0%
*Consequences of the genetic diagnosis to you*						
Inform lifestyle changes to avoid triggers of AF	625 (406–844)	< 0.00001		557 (351–762)	< 0.00001	
Inform changes in treatment of co-morbidities and other risk factors that predisposed to AF	973 (763–1182)	< 0.00001		801 (633–970)	< 0.00001	
Inform ongoing monitoring to detect other heart complications that are related to the genetic diagnosis of AF	1026 (825–1227)	< 0.00001	14.4%	812 (587–1037)	< 0.00001	12.2%
Inform changes in AF treatment and management	619 (388–849)	< 0.00001		461 (249–673)	< 0.00001	
No consequence	Base					
*Consequences of the genetic diagnosis to your family*						
Identify the risk of developing AF and initiate baseline cardiac investigation	999 (838–1160)	< 0.00001	12.0%	805 (629–982)	< 0.00001	10.3%
No consequence	Base					
Cost of testing to you (AU$)	–	–	61.4%	–	–	65.7%

Marginal WTP assesses the value that participants are willing to pay for each attribute. Importance score assesses the relative importance of each attribute on participants’ decision to take up genomic testing for AF

*AF* atrial fibrillation, *WTP* willingness to pay

#### Marginal WTP and Incremental WTP

Table 3 also presents the marginal WTP for each attribute. In general, the attributes of testing were more valued by participants in the symptomatic survey compared with those in the at-risk survey. Specifically, participants on average were willing to pay $13 (at-risk survey, $11) for every additional person in a hundred receiving a genetic diagnosis for AF; $619 (at-risk survey, $440) to gain knowledge about the future recurrence of AF and disease progression. For the clinical consequences of a genetic diagnosis for AF, compared to no consequences, participants were willing to pay $625 (at-risk survey, $557) for information on lifestyle changes to avoid triggers of AF; $973 (at-risk survey, $801) for better treatment of co-morbidities and other predisposing risk factors for AF; $1026 (at-risk survey, $812) for opportunities to initiate ongoing monitoring to detect other heart complications; and $619 (at-risk survey, $461) for improvements in AF treatment and management. If other family members could be informed about their risk for developing the condition and start some baseline cardiac investigations, the value for genomic testing in AF will on average increased by $1000 in the symptomatic sample and $805 in the at-risk sample respectively.

Table 4 reports the estimated WTP for genomic testing in AF under two scenarios in a publicly funded healthcare system, where the government reimburses the cost of testing. In both scenarios, it is assumed that with genomic testing, an additional 30% of individuals would receive a genetic diagnosis for their AF. Among those diagnosed, 50% would gain knowledge about disease progression, and family members of those receiving a diagnosis would be advised to be aware of their AF risks. The WTP for genomic testing without significant clinical implications compared with standard care is estimated to be $2863 for the symptomatic sample and $2596 for the at-risk sample (Scenario 1). In Scenario 2, WTP was examined for a genomic test that would advance treatment and management for 60% of those with a genetic diagnosis. The estimated WTP is $2973 for the symptomatic survey and $2670 for at-risk survey^3^.

**Table 4:** Incremental WTP for genomic testing relative to standard care for AF

Attributes		Incremental differences (genomic testing vs standard care)			
		Scenario 1		Scenario 2	
		Gene-specific therapy and implications for ablation strategies (not available)		Gene-specific therapy and implications for ablation strategies (available)	
Number of people who receive genetic diagnosis (out of 100)		30 more		30 more	
Knowledge about disease progression		50% more for all additional people diagnosed (i.e. 15%)		50% more for all additional people diagnosed (i.e. 15%)	
Consequences of the genetic diagnosis to you (inform changes in AF treatment and management)		–		60% more for all additional people diagnosed (i.e. 18%)	
Consequences of the genetic diagnosis to your family (identify the risk of developing AF and initiate baseline cardiac investigation)		For all additional people diagnosed (i.e. 30%)		For all additional people diagnosed (i.e. 30%)	
Cost of testing to you (AU$)		Reimbursed by the government		Reimbursed by the government	
WTP (95% CI)	Preferences for symptomatic patients	Mean: $2863 ($2443–$3282)	*P* < 0.00001	Mean: $2973 ($2556–$3391)	*P* < 0.00001
	Preferences for at-risk relatives	Mean: $2596 ($2217–$2974)	*P* < 0.00001	Mean: $2670 ($2325–$3074)	*P* < 0.00001

Incremental WTP assesses the value that participants are willing to pay for genomic testing relative to the standard care of AF

*AF* atrial fibrillation, *CI* confidence interval, *WTP* willingness to pay

#### Testing Uptake

**Fig. 2:**
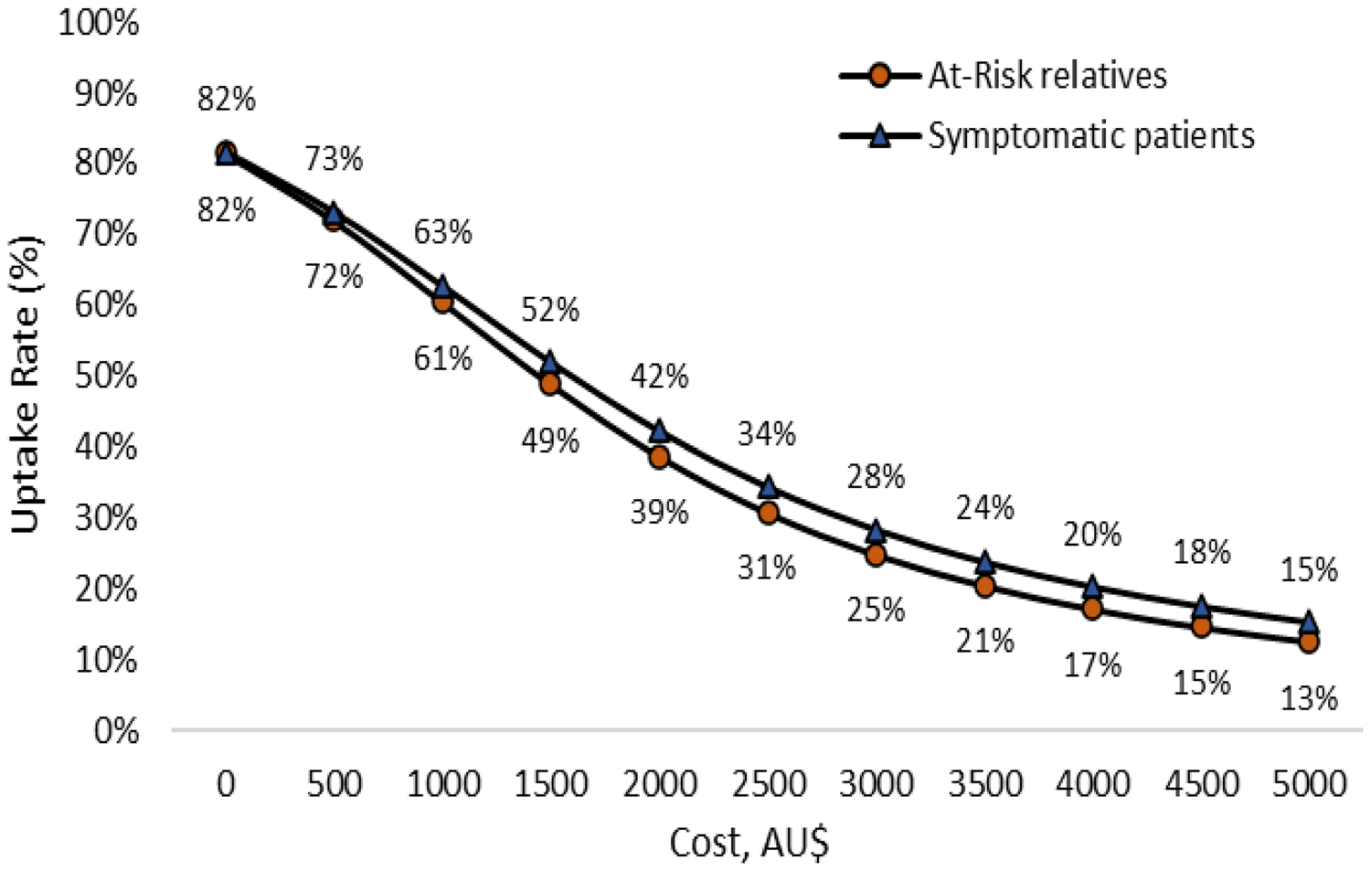
Uptake rate of genomic testing for atrial fibrillation across different out-of-pocket cost levels. Assumptions for genomic testing: additional 30 out of 100 individuals could receive a genetic diagnosis for their atrial fibrillation, gene-specific therapy and implications for ablation strategies would be available to 60% of those with a genetic diagnosis, 50% would gain knowledge about disease progression and family members of those receiving a diagnosis would be advised to be aware of their atrial fibrillation risks

Figure 2 shows the estimated uptake of genomic testing in AF across different cost levels, assuming constant additional genetic diagnosis at 30%, with implications for disease progression, potential therapeutic interventions and risk monitoring strategies for family members. Overall, testing uptake was higher among individuals in the symptomatic survey compared with the at-risk survey. If there was no out-of-pocket cost, 82% respondents were predicted to take up genomic testing. Testing uptake decreased to around 49–52% as the cost increases to $1500. Less than 30% of the respondents would opt for testing if the cost is $3000, and the uptake rates fall below 20% when the testing cost exceeds $4000.

We further presented the uptake of genomic testing for AF across different sociodemographic groups (Figs. S1 and S2 of the ESM). The results showed significant disparities in uptake across socioeconomic status groups, suggesting potential inequalities in access to genomic testing. In both surveys, individuals with relatively lower socioeconomic status (e.g. without private health insurance, lower income or less education) were less likely to take up genomic testing. Lower uptakes were also observed among older adults, those residing in non-metropolitan areas and individuals with limited knowledge of genetics. Furthermore, as the cost of testing increased, the uptake gaps between the socioeconomically advantaged and disadvantaged groups became more pronounced.

### Estimation of LC Model and Fractional Logit Model

Figures S3 and S4 of the ESM present the results of the LC analysis, which divided the sample into three distinct classes, as well as the fractional logit regression results on the associations between personal characteristics and class membership probabilities, which provide additional statistical evidence on how sociodemographic and attitudinal characteristics influence testing uptakes.

For the symptomatic survey, Class 1 represents 21% of the sample and includes individuals with a negative preference for genomic testing. Membership in Class 1 was significantly associated with older age, lower income, not having private health insurance, lack of experience with genetic conditions and poorer health. Class 2 accounts for 29% of the sample and consists of people with a positive preference across all attributes but high price sensitivity. Membership in this class was significantly linked to higher education, higher income, living in non-metropolitan areas and being more risk averse. Class 3 is the largest group, comprising half of the sample, and includes people who have a positive preference for genomic testing regardless of the clinical consequence of informing disease treatment and management. Compared with Class 2, Class 3 members were overall less price sensitive but placed a lower value on attributes. Being young, having private health insurance, personal experiences with genetic conditions and heart conditions other than AF, and taking more risks was significantly associated with membership in Class 3.

Similar patterns were observed in the at-risk survey. Class 1 (25% of the sample) had a negative preference for genomic testing, and class membership was significantly associated with no private health insurance. Class 2 (27% of the sample) had positive preferences across all attributes but were more price sensitive, with membership significantly linked to higher education, having private health insurance, experiences of AF, less knowledge about genetics and being more risk averse. Class 3 (48% of the sample) includes people with positive preferences for genomic testing regardless of the diagnosis yield. In general, Class 3 members had a lower marginal utility gain across the attributes but were less price sensitive than those in Class 2. Class 3 membership was significantly linked to having experience with genetic conditions, and a good knowledge of genetics.

## Discussion

In this study, we used a DCE to quantify the economic value of the diagnostic, prognostic, clinical and familial aspects of genomic testing for AF in the hypothetical context of symptomatic patients and at-risk relatives. Participants in both the symptomatic and at-risk surveys demonstrated positive preferences for the health and non-health outcomes associated with genomic testing. The highest values were attributed to the clinical consequence of providing opportunities for ongoing monitoring and early detection of other heart complications related to AF, followed by implications for other family members to understand their risks and initiate presymptomatic monitoring and surveillance of the condition. We found that in a publicly funded healthcare system where cost of testing is reimbursed by the government, participants in the symptomatic survey were willing to pay approximately $2900 for testing, with an expected uptake rate of 82%. The economic value of genomic testing for those in the at-risk survey was about 10% less, while their estimated uptake rate was similar. Our results also suggested significant disparities in estimated testing uptake between socioeconomically advantaged and disadvantaged groups. Individuals with a lower socioeconomic status (e.g. without private health insurance, lower income or less education) were less likely to take up genomic testing for AF. Lived experiences of genetic and heart conditions, as well as better knowledge of genetics and health risk attitudes positively influenced the decisions for taking up genomic testing for AF.

The high value that individuals placed across all components of genomic testing has been well documented in other clinical contexts. Consistent with previous research [12–14, 36], our findings highlight the significant importance that respondents attribute to improvements in medical management, as well as the information and knowledge gained about the condition and family risk through genomic testing. Goranitis et al. [12] concluded that the availability of preventive or treatment options and improvement in the medical care process were the most important attributes of genomic testing for adult-onset conditions. Similarly, studies on preferences for genomic precision medicine in Canada showed that changes in patients’ health and function from genomic testing was the most important attribute for both healthcare payers and practicing clinicians [37, 38]. Meng et al. [36] found that both the general public and parents with lived experience of severe speech disorders valued the genomic test’s capacity to improve medical care process and provide knowledge about the child’s future health and development the most. Marshall et al. [15] also found that parents of children with rare genetic diseases place the highest value on obtaining knowledge about disease cause, progression and family risk, with a WTP of US$6038 (AU$8695) for such information.

Limited evidence exists on preferences for genomic testing in cardiovascular disease and our study is the first to empirically elicit consumer preferences and values for genomic testing in AF. Our findings align with the existing evidence that individuals highly value the integration of genomic technology and services into the clinical care of cardiovascular disease. Goranitis et al. [12] assessed the WTP for genomic testing in a range of adult conditions in Australia, the study estimated that society was willing to pay $2591–$4660 for genomic testing in cardiac arrhythmias. The uptake of panel testing for inherited cardiovascular disease was estimated at 98% in the UK based on preferences from health professionals who order genomic testing [18]. The study by Kasparian et al. [19] explored parents’ preferences for paediatric cardiac genetics services and concluded that the uptake is 34–93% depending on service criteria. Johansson et al. [20] elicited the preferences of cardiopulmonary disease research participants for receiving genetic risk information from genomic sequencing. They concluded that when testing is provided free of charge and preventive measures are 90% effective, uptake for receiving genetic information is above 89%.

One interesting finding from our study is that individuals attributed a greater utility to a genomic test that informs the management of other cardiac complications and comorbidities associated with AF, rather than one that solely inform changes in AF treatment and management. Given that AF may coexist with, or increase the risk of other cardiovascular diseases, such as ventricular cardiomyopathies and heart failure [39], this finding may indicate that individuals have a stronger preference toward managing the broader spectrum of cardiovascular and non-cardiovascular comorbidities through genetic diagnosis of AF. This further highlights the broader value of genomic testing, which can inform the prevention, monitoring and management of other serious health conditions beyond AF.

We also identified that an individuals’ interest in undergoing genomic testing for AF is influenced by their personal experience of genetic and heart conditions, knowledge of genetics and attitudinal characteristics. This is consistent with existing genomic testing literature, which indicates that an individual’s understanding of genetic information may affect their risk management decisions, and that their risk attitudes and perception of risks are significant determinants of intentions to participate in genomic testing [40, 41]. Consequently, education programmes are needed to improve patients’ understanding of the risks and benefits associated with genomic testing. Additionally, our findings emphasise the potential disparity in testing uptake. Targeted initiatives, such as subsidies and tailored health education programmes, are needed to improve the access and availability of healthcare and resources to socioeconomic disadvantaged groups. Further research is required to guide the development of targeted educational resources and information materials to enhance the effectiveness of counselling strategies and support equitable implementation of genomic testing.

This is the first study to examine the societal uptake and economic value of genomic testing in AF, utilising a representative sample of adults from the general Australian population—the primary stakeholders of the healthcare system. Our study design allowed for a detailed assessment of how individuals value different clinical and non-clinical outcomes of testing, and extensive exploration of how preferences vary across the population. Given those values, our results facilitate the integration of individual preferences, values and uptake probabilities into the economic evaluation of cascade and proband testing in AF.

The findings from our study should be considered with certain limitations. First, our survey primarily focused on genomic testing for rare disease-causing variants where there is a clear binary outcome. However, there is an emerging possibility of using polygenic risk scores, which provide a graded assessment of AF susceptibility. More research is needed to explore how individuals perceive and value the risk information from polygenic risk scores, and how the values vary based on their AF risks and risk preferences. Secondly, societal preferences are likely to differ from how patients and clinicians view genomic testing for AF. Further research comparing the preferences of the general population, patients and clinicians in this context would provide valuable insights. Moreover, one key limitation in DCE methods is that respondents’ choices may not fully reflect their behaviour in real-world situations, given the hypothetical nature of DCE choice tasks. This is referred to as “hypothetical bias”, where choices made in this experimental context could differ from decisions individuals would make if they had actually developed AF symptoms or had close family members diagnosed with AF and were offered genomic tests in clinical care. However, past evidence on the external validity of DCE studies indicates that it is a reliable tool to produce reasonable predictions of individuals’ health behaviours [42]. Additionally, some relevant but lower ranked factors related to genomic testing for AF, such as incidental findings from testing, were not included in our choice tasks to minimise respondents’ cognitive burden. Our selection of attributes was informed by support from the general public and experts specialising in clinical genomics; therefore, the chosen attributes reflect the most relevant and influential aspects of the decision-making process, providing valid and reliable evidence. Finally, our findings suggest that risk attitudes and perception of risk (e.g. based on personal experiences of cardiac conditions) can impact the values individuals placed on genomic testing for AF. However, incorporating risk attitudes and perceptions into decision making in DCE may lead to deviations from the random utility theory underlying conventional discrete choice analysis, where risk is evaluated in a linear form [43]. Further research is needed to explore how to best account for risk attitudes and perceptions at both the design and analysis stages in discrete choice analysis in health decision making under uncertainty.

## Conclusions

Our study provided the first empirical evidence that the use of clinical genomic testing for AF is highly valued by the Australian general population. For both symptomatic patients and at-risk relatives, the clinical implications for monitoring other cardiac complications beyond AF, as well as risk management strategies for other family members, are the most valued aspects. This evidence provides useful insights for prioritising these elements in the translation of genomic testing into clinical practice for AF. In addition, it is pertinent to develop counselling strategies tailored to individuals’ sociodemographic and attitudinal characteristics to maximise the testing uptake and provide more equitable access to testing, especially for the socioeconomically disadvantaged groups. Further research is needed to quantify how psychological and behavioural factors influence decisions to take up genomic testing, and to examine how risk attitudes and perceptions can be incorporated to better inform and optimise the implementation of genomic testing in the healthcare system.

## Supplementary Information

Below is the link to the electronic supplementary material.

Supplementary file1 (DOCX 76 KB)

Supplementary file2 (DOCX 828 KB)

## Footnotes

1 We incorporated interaction terms between each attribute and the two contexts (symptomatic patients and at risk). Priors for the interaction terms were informed by published studies and expectations from the research team.

2 Beyond the standard data quality checks applied by Pureprofile, we included a traffic-light attention check question in the survey, and participants who failed this question (*n* = 8) were excluded from the primary analysis.

3 We also computed the WTP values derived from the population-simulated data. The simulated WTP values were closely aligned with the mean estimates and fell within their CIs, which provided robustness of our WTP estimates.

## Declarations

### Availability of Data and Material

The de-identified dataset generated and analysed in the study can be made available from the corresponding author on reasonable request.
