# Supplementary material for: Societal Preferences, Values and Priorities for Genomic Testing for Atrial Fibrillation: Evidence from Two Discrete Choice Experiments
Source: Patient. 2026 Feb 13;19(4):547–59. doi: 10.1007/s40271-026-00801-w (PMC13287222; doi:10.1007/s40271-026-00801-w)
Supplement: Supplementary file 1 — Supplementary file1 (DOCX 76 KB) [file 40271_2026_801_MOESM1_ESM.docx]

**Understanding preferences around genomic testing for Atrial Fibrillation**

We are interested in understanding society’s views on the importance of the information and benefits that genomic testing could offer to people experiencing Atrial fibrillation (AF) and their families. As a member of the Pureprofile panel, we are inviting you to do a survey about genomic testing for finding an explanatory cause for AF.

Genomic tests examine our genes. Genes contain information that makes us who we are, including our eye and hair colour. Genomic testing can be used to screen a range of cardiac-related genes to detect signs of AF or risk factors for the development of AF. AF is a heart condition in which the top chamber of your heart (the atria) beat fast and irregularly. In AF, your heart may not pump blood around the body properly. If left untreated, AF may lead to serious health problems, such as stroke and heart failure.

Families who have members with AF, can benefit from genomic testing, as it is possible that there is a genetic cause to AF. Getting a genomic result in people who are experiencing AF may help to explain the causes of AF and to better target treatment and prevention. There are also implications for other family members, as some of them are likely to carry the same genetic risk variant and develop AF in the future.

**With this survey, we want to understand your views about the importance of the different information and benefits that genomic testing may provide to people diagnosed with AF or suspected of having AF, and their families.**

The survey will take about 15 minutes to complete.

Your participation is voluntary. Your responses will be confidential and will not be shared with anyone outside the research team. This research has ethical approval from the Medicine and Dentistry Human Ethics Committee of the University of Melbourne. You can withdraw from this research at any time and without any consequences.

In the following page, we provide a description of the project and the nature of your participation.

To enter the survey, you will need to provide your consent. Your consent will allow us to use the information you provide for our research.

If you have any questions, please contact A/Prof Ilias Goranitis ([ilias.goranitis@unimelb.edu.au](mailto:ilias.goranitis@unimelb.edu.au)) or Dr Cun Liu (cun.liu@unimelb.edu.au).

We appreciate your time and support.

**Plain Language Statement**

Centre for Health Policy/ Melbourne School of Population and Global Health

***Project: Understanding preferences around genomic testing for Atrial Fibrillation***

A/Prof Ilias Goranitis Email: [ilias.goranitis@unimelb.edu.au](mailto:ilias.goranitis@unimelb.edu.au)

Dr Cun Liu Email: [cun.liu@unimelb.edu.au](mailto:cun.liu@unimelb.edu.au)

**Introduction**

We are contacting you because you are a member of the Pureprofile panel. The following few pages will provide you with further information about the project, so that you can decide if you would like to take part in this research.

Please take the time to read this information carefully. You may ask questions about anything you don’t understand or want to know more about. Your participation is voluntary. If you don’t wish to take part, you don’t have to. If you begin participating, you can also stop at any time. This research is funded by a National Heart Foundation of Australia Predictive Modelling Grant titled “Investigating the role of genetics for risk stratification in Atrial Fibrillation”.

**What is this research about?**

Genomic testing can be used to test changes in a range of cardiac-related genes at one time to find a diagnosis of Atrial Fibrillation (AF) or identify the risk of developing AF. Applying genomic technologies in the AF setting can offer significant benefits, including providing a diagnosis, informing treatment, and enhancing knowledge about the condition. This may come with potential challenges, such as anxiety or uncertainty in cases where AF will not be resolved or another health condition is diagnosed.

With this research, we want to understand the importance that people in the society, who may or may not have a lived experience of AF, place on the information and benefits that genomic testing can provide to people who are likely to develop AF or have been diagnosed with AF, as well as their families. This will help us to identify how important society believes these benefits are to people affected by AF and their families.

**What will I be asked to do?**

If you agree to participate, you will be asked to complete one survey. The survey will take about 30 minutes to complete. Initially, the survey begins by asking questions about you and your experiences with genomic tests and AF. The survey then describes the information and benefits that people hope to get from genomic testing for AF. You will then be asked to make choices based on how important these benefits are to you.

**What are the possible benefits?**

There will be no direct benefits to you from your participation. However, there are great potential benefits for society. Understanding what genomic test characteristics matter to people will allow the best implementation of genomics in the Australian health system and beyond. Standard incentives for survey participation will be provided per Pureprofile protocols.

**What are the possible risks?**

We do not anticipate any risks in participating in this study. It may be that taking part may cause you to think about your or your families’ wellbeing. If you have any concerns about your health, please seek advice from your GP.

**Do I have to take part?**

No. Participation is completely voluntary, and you can withdraw at any time and without any consequences. Please note that once you complete the survey, it is not possible to withdraw from the study because data are anonymised and not identifiable.

**Will I hear about the results of this project?**

A summary of the survey findings will be placed on the University of Melbourne and the Victor Chang Cardiac Research Institute websites. We will also be sharing the results of the survey more widely via academic journals and conferences, and through the development of a briefing to distribute to patient advocacy groups, policy makers, and health providers.

**What will happen to information about me?**

All the information gathered from the surveys will be treated confidentially. Survey data will be held on University of Melbourne computers and retained for 5 years following the publication of our findings.

**Who is funding this project?**

This project is being funded by the National Heart Foundation of Australia through the Predictive Modelling Grant. The funding will contribute to survey distribution. The funding agreement ensured researchers’ independence in designing the study, conducting the research, interpreting the data, writing, and publishing the findings.

**Where can I get further information?**

If you would like more information about the project, please contact the researchers;

A/Prof Ilias Goranitis Email: [ilias.goranitis@unimelb.edu.au](mailto:ilias.goranitis@unimelb.edu.au)

Dr Cun Liu Email: cun.liu@unimelb.edu.au

You can keep a copy of this form for your records.

**Who can I contact if I have any concerns about the project?**

This project has human research ethics approval from The University of Melbourne (Project ID Number: 12250). If you have any concerns or complaints about the conduct of this research project, which you do not wish to discuss with the research team, you should contact the Research Integrity Administrator, Office of Research Ethics and Integrity, University of Melbourne, VIC 3010. Tel: +61 3 8344 1376 or Email: research-integrity@unimelb.edu.au. All complaints will be treated confidentially. In any correspondence please provide the name of the research team and/or the name or ethics ID number of the research project.

**Consent**

Do you consent to participate in this survey as outlined in the Plain Language Statement?

By clicking yes, you provide your consent to use survey responses for the purposes of our study.

*Please remember that you can withdraw from the study at any point before the survey is completed. As soon as the survey is completed, we are not able to identify your responses because the data are anonymised and not identifiable.*

☐ Yes, I consent to participate in this survey as outlined in the Plain Language Statement

☐ No, I do not wish to consent to participate in this survey

☐ I would like further information about this survey

**Section 1**

**Please complete the following section to tell us about yourself:**

1. How old are you? _________________ years
2. What is your gender?

☐ Female ☐ Male ☐ Prefer to self-describe (please specify):___________

1. What is your current marital status?

☐ Never married

☐ De facto (living with a partner)

☐ Married

☐ Widowed

☐ Divorced/separated

☐ Other (please specify): _________________

1. What is your highest level of education?

☐ Year 11 or below

☐ Year 12 or equivalent

☐ Certificate

☐ Diploma/advanced diploma

☐ Bachelor’s degree

☐ Graduate diploma/certificate

☐ Post-graduate degree

☐ Other (please specify): _________________

1. What is your household’s annual gross (before tax) income?

☐ Lower than $40,000 per year

☐ $40,000 - $60,000 per year

☐ $60,000 - $80,000 per year

☐ $80,000 - $100,000 per year

☐ $100,000 - $120,000 per year

☐ $120,000 - $140,000 per year

☐ $140,000 - $160,000 per year

☐ Over $160,000 per year

1. Do you have private health insurance?

☐ Yes

☐ No

1. Which of the following best describes where you live?

| New South Wales |
| --- |
| Queensland |
| Western Australia |
| South Australia |
| Victoria |
| Tasmania |
| The Australian Capital Territory |
| The Northern Territory |
| Outside Australia |
| Prefer not to answer |

1. What is your place of residence?

☐ Metropolitan

☐ Non-metropolitan

1. How many children do you have? _______
2. How many of your children are 15 years or younger? ______

**Experiences with genetic conditions**

1. How familiar do you feel you are with how genetic conditions affect people’s lives and the lives of those around them?

☐ Very familiar

☐ Moderately familiar

☐ Slightly familiar

☐ Not at all familiar

1. Do you or any of your close family members or friends have a genetic condition?

☐ Yes

☐ No

1. Have you or any of your close family members or friends ever had a genetic or genomic test?

☐ Yes

☐ No

1. Have you read or heard about genomic testing before receiving this questionnaire?

☐ Yes

☐ No

**Knowledge about genetics**

1. How knowledgeable do you feel you are about genetics?

☐ Know it well

☐ Know a fair amount

☐ Know a little

☐ Heard of it

☐ Never heard of it

**Experiences with Atrial Fibrillation**

1. Have you ever been diagnosed by a healthcare professional with Atrial Fibrillation?

☐ Yes

☐ No

1. Have any of your close family members or friends ever been diagnosed by a healthcare professional with Atrial Fibrillation?

☐ Yes

☐ No

1. Have you ever been diagnosed by a healthcare professional with other heart conditions (e.g., stroke, heart failure)?

☐ Yes

☐ No

1. Have any of your close family members or friends ever been diagnosed by a healthcare professional with other heart conditions (e.g., stroke, heart failure)?

☐ Yes

☐ No

1. What is your height? _________cm
2. What is your weight? _________kg
3. Have you had one or more of the following which may increase the risk for Atrial Fibrillation? **(Select at least one option)**

☐ High blood pressure

☐ Sleep apnoea

☐ Diabetes

☐ Drinking alcohol (Please specify number of standard drinks you have per week): _______

☐ Smoking (Please specify number of cigarettes you smoke per day): __________

☐ Angina/heart attack/hardening of the arteries

☐ Heart valve abnormalities

☐ Other heart conditions (Please specify): ___________

☐ None of the above

**Health**

1. How is your own health today?

Give a number between 0 (worst health you can imagine) to 10 (best health you can imagine): __________

**Attitudes towards risk**

1. In general, people often face risks when making financial, career, health, or other life decisions. Overall, how comfortable do you feel you are in taking risks regarding your health?

Give a number between 0 (I am not comfortable at all) to 10 (I am extremely comfortable): __________

To proceed to the next section, please select the order of the 3 colors from the image below (from top to bottom).

| **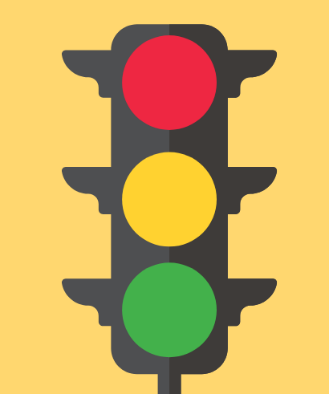** |
| --- |
| ☐ Green, Red, Orange. |
| ☐ Orange, Green, Red. |
| ☐ Red, Orange, Green. |
| ☐ Red, Green, Orange |

**Section 2**

| **What is Atrial Fibrillation?** |
| --- |

Atrial Fibrillation (AF) is the most common type of heart rhythm disorder. One in three individuals are at risk of developing AF during their lifetime, and around half a million Australians are living with AF.

AF is a heart condition in which the top chambers of your heart (the atria) beat fast and irregularly. The rapid heart rate triggered by AF can increase pressure on the heart and weaken the heart, which can lead to cardiomyopathy (i.e., a heart muscle disease that prevents the heart from pumping blood around the body properly) and heart failure. People with AF are three times more likely to develop heart failure than those without.

AF will increase the chance of blood pooling in the heart and forming clots, which can cause stroke. In Australia, AF is a major cause of stroke. People who have AF are five times more likely to have a stroke than those who do not.

Some people with AF experience palpitations, dizziness, tiredness, or shortness of breath. However, sometimes people have AF and don’t know it because they have no symptoms.

| **What causes Atrial Fibrillation?** |
| --- |

AF affects men and women and can occur at any age. However, the risk of having AF increases as you get older, and if you are overweight, smoke, have high blood pressure, high cholesterol, diabetes, or disrupted sleep. In some people, too much alcohol or caffeine can trigger an episode of AF. A person's genetic makeup can also affect whether or not AF will occur in some cases.

AF may start with short episodes that come and go and stop within 48 hours. For some people, these episodes can become more frequent or last longer, and medications can become less effective and stop working, which makes their risk of developing heart failure and stroke even higher.

| **What treatments are available for Atrial Fibrillation?** |
| --- |

Treatment for AF may involve medications and therapy to restore and keep the heart rhythm, control symptoms, and prevent serious complications such as heart failure or stroke. More specific treatments may include:

- Pharmacological therapy - Medications to stop the heart from beating too fast or restore the heart's rhythm to normal.
- Electrical cardioversion - An electric shock to the chest to reset the heart and restore the heart’s rhythm to normal.
- Blood clot prevention - Medications to thin the blood to prevent the formation of blood clots that cause strokes.
- Pacemaker - A small insertable device that sends electrical signals to help control the heartbeat.
- Catheter Ablation – A procedure that makes a small scar in the heart to block faulty electrical impulses.

Here is a video of a patient with AF.

<https://www.youtube.com/watch?v=uYbOGhzpPUw&t=6s>

**Section 3**

Genomic tests examine our genes. Genomic testing can investigate a range of genes associated with cardiac disorders at one time with the aim of finding a diagnosis of AF or identifying the risk of developing AF in the future. Genomic testing may have a range of outcomes that are valued by individuals experiencing AF and their families. This might include: identifying a genetic cause for AF, understanding the risk of developing AF in family members, informing treatment and family planning, allowing access to clinical trials, and contributing to research, policy and society. However, a genetic diagnosis may bring challenges, such as concern over genetic ‘label’, potential disclosure of the results to life insurers, or potential unexpected diagnosis of another health condition.

This section lists information about five characteristics of genomic testing that matter to individuals and families affected by AF. Please read carefully as the next section will ask you to make choices based on how important these are to you.

**Characteristic 1**

**Number of people who receive a genetic diagnosis**

This characteristic tells you how likely it is that there is a genetic cause for AF. For example, “20 out of 100” means that for every 100 people with similar problems being tested, 20 will have a genetic cause for their AF and 80 will not.

| Please note:  Our understanding of genetic conditions is improving all the time. Even when a diagnosis is not made at the time of testing, the genetic data may be reanalysed in future when new analysis methods come to hand and a diagnosis maybe made at a later date. |
| --- |

**Characteristic 2**

**Knowledge about the future recurrence of AF and disease progression**

This characteristic tells you whether a genomic diagnosis can enable health professionals to provide you with additional information about AF recurrence and disease progression. This information would not be able to be obtained in the absence of a genomic diagnosis.

In some cases, knowing the genetic cause for AF can provide specific information about whether episodes of AF will happen repeatedly over time. Sometimes, identifying a genetic cause may reveal that there will be some changes in your atrium structure or function, and your AF is likely to be accompanied by other heart complications, such as hypertrophic cardiomyopathy (a heart condition in which your heart muscle become thickened). The knowledge gained from the genomic result may be perceived either positively or negatively regarding the individual’s future health development.

**Characteristic 3**

**Consequences of the genetic diagnosis to you**

This characteristic tells you the likely preventive or treatment options available to improve your health once a genetic cause to AF is identified.

Sometimes, there might be no options available. Other times, if a genetic change that causes AF is found, you are likely to have continuous AF episodes. For example, you may have abnormal heart beats daily for several minutes or hours that last for few days in a row, and your heart rhythm may not be able to be restored to normal without treatment. This is different to people who have an acute trigger for AF, such as an episode triggered by drinking a lot of alcohol in one session, which can be avoided or reversed. Individuals with a genetic cause for their AF may need to:

- work extra hard to make lifestyle changes to lower the chance of triggering an AF episode, such as watching blood pressure, limiting alcohol and coffee intake, avoiding smoking or losing weight.
- proactively work on the treatment of other health conditions (e.g., high blood pressure and diabetes) related to AF.

Changes in genes linked to AF can affect a person’s likelihood of developing other heart conditions. A genetic diagnosis of AF may also reveal your risk of developing other heart complications in the future (e.g., dilated cardiomyopathy - a heart muscle disease that causes the heart chamber/ventricle to grow large and become weaker). In some cases, individuals identified as having a genetic cause for their AF may need ongoing surveillance to detect these heart problems.

Individuals with a genetic cause for AF may not react to a treatment, respond to a treatment differently, or have recurrence of AF after treatment. A genomic result of AF may be useful for predicting the efficacy of ablation or drug therapy, allowing for possible changes in treatment options.

| Please note:  Although there might be improvements in your health with the options provided, the extent to which these improvements will change your AF outcomes in the future is uncertain. |
| --- |

**Characteristic 4**

**Consequences of the genetic diagnosis to your family**

This characteristic tells you the likely implications to other family members from a genomic result of AF. Following the genetic diagnosis of AF, other family members can learn about their risk of developing AF, become more aware of AF symptoms, and get checked out if they have any symptoms, which may prompt earlier diagnosis and medical treatment.

**Characteristic 5**

**Cost of testing to you**

Genomic testing is currently available for many types of heart disease but not AF. If such a test was to become available, the Federal Government does not pay for it. This characteristic tells you how much you would need to pay out-of-pocket for the test. The cost could range from a few hundred dollars up to several thousand dollars. The test will be performed once and the payment will be a one-off payment.

| **Please note:**  The survey does not require you to actually pay for the test.  To help us accurately value the test, please pay attention to the actual costs of the test presented to you and carefully consider whether this is an amount you would be willing to pay if your doctor recommended a genomic test.  Remember that this amount would no longer be available for you to spend on other things, such as grocery or utility bills. |
| --- |

**Section 4**

**In this section, we want to understand how important each of these characteristics is to you.**

**Please imagine that you have developed symptoms of Atrial Fibrillation (AF) that started to concern you. Your doctor has conducted some initial investigations and recommends a genomic test to explore if there is an underlying genetic cause for AF.**

We will present 13 scenarios to you. Within each scenario, there will be two different situations: ‘Situation 1’ and ‘Situation 2’. The two situations will differ in five of the characteristics described earlier.

You will be asked to select the situation under which you would prefer to have the genomic test. If you would prefer to have the test in both situations, please select the situation where you think genomic testing would be most beneficial. If you would not choose to have the test under either of these situations, please select the option ‘Neither’.

| Please note:  **While completing the remainder of the survey, please remember to imagine that** **you have developed symptoms of AF that started to concern you. Your doctor has conducted some initial investigations and recommends a genomic test to explore if there is an underlying genetic cause for AF.** |
| --- |

**An example**

**Under which situation would you like to have a genomic test? You can choose either ‘Situation 1’, ‘Situation 2’, or ‘Neither’ (i.e., you would not like to have a genomic test).**

The person who answered the question below decided that genomic testing would be the most beneficial in Situation 1. The decision was made by comparing the characteristics of Situation 1 with those of Situation 2. This person considered that the additional cost involved in Situation 1 was worth the benefit gained from the remaining characteristics.

Please note that in surveys like this, it has been found that some people tend to overestimate or underestimate how much they would really be willing to pay. Thus, they may choose situations that they would not actually prefer in real life. It is important that your choices here are realistic. If you would like to remind yourself during the survey what each of these characteristic mean, point/click on the characteristics of interest and a pop-up box with the description will appear.

|  | **Situation 1** | **Situation 2** | **Neither** |
| --- | --- | --- | --- |
| Number of people who receive a genetic diagnosis | 35 out of 100 | 5 out of 100 |  |
| Knowledge about future recurrence of AF and disease progression | Yes | No |  |
| Consequences of the genetic diagnosis to you | Inform changes in AF treatment and management | Inform lifestyle changes to avoid triggers of AF |  |
| Consequences of the genetic diagnosis to your family | Identify the risk of developing AF and initiate baseline cardiac investigation | No consequence |  |
| Cost of testing to you | A$3000 | A$500 |  |
| **Under which situation would you like to have a genomic test** |  |  |  |

**Enter choice experiment**

Choice tasks are available upon request

**Feedback**

1. How did you find the questions in the survey?

| ☐ Easy | ☐ Moderate | ☐ Difficult |
| --- | --- | --- |

1. Have you got any comments about this questionnaire that you would like to share with us?

__________________________________

**Acknowledgement**

**Thank you for taking the time to complete our survey.**
