# Supplementary material for: Societal Preferences, Values and Priorities for Genomic Testing for Atrial Fibrillation: Evidence from Two Discrete Choice Experiments
Source: Patient. 2026 Feb 13;19(4):547–59. doi: 10.1007/s40271-026-00801-w (PMC13287222; doi:10.1007/s40271-026-00801-w)
Supplement: Supplementary file 2 — Supplementary file2 (DOCX 828 KB) [file 40271_2026_801_MOESM2_ESM.docx]

**Table S1.** Participant characteristics

|  | **Symptomatic (N=503)** | | **At Risk (N=506)** | |
| --- | --- | --- | --- | --- |
|  | **Number** | **Percentage** | **Number** | **Percentage** |
| **Age** | Mean = 48; SD = 17 | | Mean=48; SD=17 | |
| **Gender (Female)** | 252 | 50.1 | 267 | 52.8 |
| **Marital status** | | | | |
| Married or in a de facto relationship | 313 | 62.2 | 320 | 63.2 |
| Other | 190 | 37.8 | 186 | 36.8 |
| **Educational status ( Higher education)** | 218 | 43.3 | 237 | 46.8 |
| **Annual household income** | | | | |
| Lower than AU$40,000 per year | 74 | 14.7 | 73 | 14.4 |
| AU$40,000 - AU$60,000 per year | 83 | 16.5 | 77 | 15.2 |
| AU$60,001 - AU$80,000 per year | 56 | 11.1 | 68 | 13.4 |
| AU$80,001 - AU$100,000 per year | 74 | 14.7 | 59 | 11.7 |
| AU$100,001 - AU$120,000 per year | 51 | 10.1 | 56 | 11.1 |
| AU$120,001 - AU$140,000 per year | 41 | 8.2 | 42 | 8.3 |
| AU$140,001 - AU$160,000 per year | 47 | 9.3 | 43 | 8.5 |
| Over AU$160,000 per year | 77 | 15.3 | 88 | 17.4 |
| **State** | | | | |
| New South Wales | 154 | 30.6 | 154 | 30.4 |
| Queensland | 103 | 20.5 | 99 | 19.6 |
| Western Australia | 44 | 8.8 | 48 | 9.5 |
| South Australia | 38 | 7.6 | 37 | 7.3 |
| Victoria | 139 | 27.6 | 140 | 27.7 |
| Tasmania | 13 | 2.6 | 14 | 2.8 |
| The Australian Capital Territory | 9 | 1.8 | 10 | 2 |
| The Northern Territory | 3 | 0.6 | 4 | 0.8 |
| **Metropolitan** | 362 | 72 | 368 | 72.7 |
| **Number of Children > 0** | 330 | 65.6 | 340 | 67.2 |
| **Private health insurance (Yes)** | 318 | 63.2 | 349 | 69 |
| **Familiarity with the impact of genetic conditions** | | | | |
| Very familiar | 74 | 14.7 | 70 | 13.8 |
| Moderately familiar | 200 | 39.8 | 225 | 44.5 |
| Slightly familiar | 174 | 34.6 | 164 | 32.4 |
| Not at all familiar | 55 | 10.9 | 47 | 9.3 |
| **Experience of a genetic condition** | 183 | 36.4 | 201 | 39.7 |
| **Experience of a genetic or genomic test** | 121 | 24.1 | 119 | 23.5 |
| **Knowledge of genetics** | | | | |
| Know it well | 16 | 3.2 | 15 | 3 |
| Know a fair amount | 88 | 17.5 | 85 | 16.8 |
| Know a little | 249 | 49.5 | 261 | 51.6 |
| Heard of it | 128 | 25.5 | 127 | 25.1 |
| Never heard of it | 22 | 4.4 | 18 | 3.6 |
| **Experience of AF** | 107 | 21.3 | 102 | 20.2 |
| **Experience of other heart conditions** | 268 | 53.3 | 292 | 57.7 |
| **Heard about genomic testing before** | 256 | 50.9 | 255 | 50.4 |
| **Health score (0-10)** | Mean = 6.7; SD=1.7 | | Mean=6.8; SD=1.6 | |
| **Health risk attitudes (0-10)** | Mean = 5.4; SD=2.4 | | Mean=5.6; SD=2.4 | |

SD indicates standard deviation.

**Table S2.** Mixed Logit Regression with Main Attributes and Individual Characterisitcs

| Attributes |  | Symptomatic (n=503) | P value | At risk (n=506) | P value |
| --- | --- | --- | --- | --- | --- |
| Number of people who receive a genetic diagnosis | Mean | 0.012 (0.006 to 0.018) | <0.00001 | 0.014 (0.009 to 0.02) | <0.00001 |
|  | SD | 0.034 (0.027 to 0.04) | <0.00001 | 0.03 (0.023 to 0.036) | <0.00001 |
| Knowledge about future recurrence of AF and disease progression (Yes) | Mean | 0.548 (0.44 to 0.656) | <0.00001 | 0.454 (0.355 to 0.553) | <0.00001 |
|  | SD | 0.476 (0.338 to 0.614) | <0.00001 | 0.382 (0.248 to 0.515) | <0.00001 |
| Consequences of the genetic diagnosis to you | | | | | |
| Inform lifestyle changes to avoid triggers of AF | Mean | 0.643 (0.449 to 0.836) | <0.00001 | 0.52 (0.332 to 0.709) | <0.00001 |
|  | SD | 0.76 (0.523 to 0.997) | <0.00001 | 0.13 (-0.449 to 0.708) | 0.6606 |
| Inform changes in treatment of co-morbidities and other risk factors that predisposed to AF | Mean | 0.91 (0.723 to 1.097) | <0.00001 | 0.76 (0.594 to 0.927) | <0.00001 |
|  | SD | 0.336 (-0.038 to 0.709) | 0.0784 | 0.37 (0.02 to 0.719) | 0.0384 |
| Inform ongoing monitoring to detect other heart complications that are related to the genetic diagnosis of AF | Mean | 1.095 (0.905 to 1.285) | <0.00001 | 0.922 (0.709 to 1.136) | <0.00001 |
|  | SD | 0.203 (-0.319 to 0.725) | 0.4467 | 0.351 (0.006 to 0.695) | 0.0459 |
| Inform changes in AF treatment and management | Mean | 0.727 (0.511 to 0.943) | <0.00001 | 0.704 (0.489 to 0.919) | <0.00001 |
|  | SD | 0.248 (-0.3 to 0.795) | 0.3755 | 0.648 (0.382 to 0.915) | <0.00001 |
| No consequence | Base | | | | |
| Consequences of the genetic diagnosis to your family | | | | | |
| Identify the risk of developing AF and initiate baseline cardiac investigation | Mean | 0.918 (0.78 to 1.057) | <0.00001 | 0.888 (0.734 to 1.041) | <0.00001 |
|  | SD | 0.968 (0.833 to 1.102) | <0.00001 | 1.065 (0.918 to 1.211) | <0.00001 |
| No consequence | Base | | | | |
| Cost of testing to you (AU$'1000s) | Mean | -1.19 (-1.27 to -1.12) | <0.00001 | -1.31 (-1.41 to -1.21) | <0.00001 |
|  | SD | 1.19 (1.12 to 1.27) | <0.00001 | 1.31 (1.21 to 1.41) | <0.00001 |
| Genomic testing constant | Mean | -1.148 (-3.276 to 0.979) | 0.2902 | 2.203 (-0.036 to 4.442) | 0.0538 |
| Individual characteristics | | | | | |
| Aged 45+ years | Mean | -1.666 (-2.592 to -0.74) | 0.0004 | -1.173 (-2.172 to -0.173) | 0.0214 |
| Gender (Female or non-binary) | Mean | -0.716 (-1.528 to 0.096) | 0.084 | -1.483 (-2.397 to -0.569) | 0.0015 |
| Marital status (Married or in a de facto relationship) | Mean | 0.812 (-0.151 to 1.774) | 0.0982 | 1.413 (0.403 to 2.424) | 0.0061 |
| Educational status ( Higher education) | Mean | 0.273 (-0.783 to 1.33) | 0.6123 | 0.778 (-0.526 to 2.081) | 0.2422 |
| Annual household income (Over AU$120,000) | Mean | -0.012 (-1.063 to 1.038) | 0.9819 | -0.418 (-1.491 to 0.654) | 0.4448 |
| Children (Yes) | Mean | 1.136 (0.165 to 2.108) | 0.0219 | -0.084 (-1.096 to 0.929) | 0.8712 |
| Private health insurance (Yes) | Mean | 0.975 (0.114 to 1.836) | 0.0264 | 1.029 (0.042 to 2.016) | 0.0411 |
| Metropolitan (Yes) | Mean | 0.13 (-0.782 to 1.041) | 0.7805 | 0.244 (-0.704 to 1.191) | 0.6141 |
| Experience of a genetic condition | Mean | 0.116 (-0.921 to 1.153) | 0.8268 | 0.563 (-0.463 to 1.589) | 0.2819 |
| Experience of AF | Mean | 0.411 (-0.7 to 1.523) | 0.4683 | -0.185 (-1.411 to 1.041) | 0.7675 |
| Experience of other heart conditions | Mean | 0.428 (-0.528 to 1.384) | 0.3806 | -0.386 (-1.35 to 0.577) | 0.432 |
| Knowledge of genetics (Very well, Fair amount) | Mean | 0.005 (-1.185 to 1.195) | 0.9936 | 0.865 (-0.607 to 2.336) | 0.2494 |
| Health score (0-10) | Mean | 0.163 (-0.091 to 0.417) | 0.2088 | -0.058 (-0.315 to 0.198) | 0.6572 |
| Health risk attitudes (0-10) | Mean | 0.199 (0.029 to 0.369) | 0.0216 | -0.036 (-0.249 to 0.178) | 0.7434 |

Mixed logit regression analysis to assess the marginal utility with each attribute and preference heterogeneity among participants. All parameters associated with attributes are estimated as random parameters${(\beta}_{k,i}=\beta_{k}+\sigma_{k}v_{k,i})$ . The mean ($\beta_{k}$) and standard deviation (SD, $\sigma_{k}$) associated with the specified distribution $v_{k,i}$ were estimated. The mean ($\beta_{k}$) represents the average marginal utility over the sampled population, while the SD ($\sigma_{k}$) reflects the dispersion around the mean. A significant SD (P<0.1)indicates preference heterogeneity within the sampled population (i.e., different individuals have specific parameter estimates that may be different from the mean). For the cost of testing attribute, a constrained triangular distribution ($V_{i}\sim T[-1,0]$) was applied to ensure that participants have a negative preference for extra cost associated with genomic testing. A normal distribution was assumed for the other attributes ($V_{i}\sim N[0,1]$). Genomic testing constant and parameters related to individual characteristics are estimated as non-random parameters (i.e., all individuals within the sample have the same parameter estimates as the mean).

**Table S3.** Latent Class Regressions for Symptomatic Patients Survey

| Attributes | Symptomatic (n=503) | | | | | |
| --- | --- | --- | --- | --- | --- | --- |
|  | Class 1 (21%, n ≈ 105) | P value | Class 2 (29%, n≈145) | P value | Class 3 (50%, n ≈ 250) | P value |
| Number of people who receive a genetic diagnosis | 0.019 (-0.007 to 0.046) | 0.1512 | 0.009 (0.001 to 0.017) | 0.0221 | 0.008 (0.005 to 0.012) | <0.00001 |
| Knowledge about future recurrence of AF and disease progression (Yes) | 0.726 (0.079 to 1.373) | 0.0278 | 0.446 (0.246 to 0.646) | <0.00001 | 0.301 (0.221 to 0.38) | <0.00001 |
| Consequences of the genetic diagnosis to you | | | | | | |
| Inform lifestyle changes to avoid triggers of AF | 0.3 (-0.95 to 1.549) | 0.6383 | 0.327 (-0.011 to 0.665) | 0.0577 | 0.269 (0.107 to 0.43) | 0.0011 |
| Inform changes in treatment of co-morbidities and other risk factors that predisposed to AF | 0.679 (-0.425 to 1.784) | 0.2278 | 0.584 (0.255 to 0.913) | 0.0005 | 0.367 (0.206 to 0.527) | <0.00001 |
| Inform ongoing monitoring to detect other heart complications that are related to the genetic diagnosis of AF | 0.731 (-0.539 to 2.001) | 0.2591 | 0.94 (0.577 to 1.303) | <0.00001 | 0.455 (0.288 to 0.622) | <0.00001 |
| Inform changes in AF treatment and management | -0.148 (-1.698 to 1.403) | 0.8519 | 0.718 (0.364 to 1.072) | <0.00001 | 0.119 (-0.052 to 0.29) | 0.1733 |
| No consequence | Base | | | | | |
| Consequences of the genetic diagnosis to your family | | | | | | |
| Identify the risk of developing AF and initiate baseline cardiac investigation | 0.658 (-0.021 to 1.337) | 0.0574 | 0.918 (0.703 to 1.134) | 0.0001 | 0.529 (0.442 to 0.617) | <0.00001 |
| No consequence | Base | | | | | |
| Cost of testing to you (AU$`1000s) | -0.85 (-1.16 to -0.54) | <0.00001 | -1.42 (-1.55 to -1.28) | <0.00001 | -0.28 (-0.32 to -0.24) | <0.00001 |
| Genomic testing constant | -3.879 (-5.33 to -2.428) | <0.00001 | 0.784 (0.45 to 1.118) | <0.00001 | 2.716 (2.405 to 3.028) | <0.00001 |

Latent class regression analysis to split individuals into a finite number of groups with distinct preferences for genomic testing in AF.

**Table S4**. Fractional Logit Regression for Symptomatic Patients Survey.

| Individual characteristics | Symptomatic (n=503) | | | | | |
| --- | --- | --- | --- | --- | --- | --- |
|  | Class 1 (21%, n ≈ 105) | P value | Class 2 (29%, n≈145) | P value | Class 3 (50%, n ≈ 250) | P value |
| Aged 45+ years | 0.69 (0.143 to 1.236) | 0.013 | 0.273 (-0.142 to 0.689) | 0.198 | -0.635 (-1.039 to -0.231) | 0.002 |
| Gender (Female or non-binary) | 0.088 (-0.384 to 0.56) | 0.715 | 0.182 (-0.199 to 0.564) | 0.35 | -0.2 (-0.573 to 0.172) | 0.291 |
| Marital status (Married or in a de facto relationship) | -0.387 (-0.948 to 0.173) | 0.176 | 0.093 (-0.344 to 0.53) | 0.677 | 0.159 (-0.27 to 0.588) | 0.468 |
| Educational status ( Higher education) | -0.153 (-0.651 to 0.345) | 0.548 | 0.356 (-0.055 to 0.766) | 0.089 | -0.237 (-0.638 to 0.164) | 0.246 |
| Annual household income (Over AU$120,000) | -0.988 (-1.643 to -0.333) | 0.003 | 0.402 (-0.028 to 0.831) | 0.067 | 0.101 (-0.333 to 0.534) | 0.649 |
| Children (Yes) | 0.016 (-0.584 to 0.616) | 0.959 | -0.271 (-0.728 to 0.185) | 0.244 | 0.219 (-0.22 to 0.657) | 0.328 |
| Private health insurance (Yes) | -0.482 (-0.983 to 0.018) | 0.059 | -0.104 (-0.525 to 0.317) | 0.629 | 0.428 (0.016 to 0.841) | 0.042 |
| Metropolitan (Yes) | 0.418 (-0.097 to 0.932) | 0.111 | -0.376 (-0.8 to 0.048) | 0.082 | 0.1 (-0.322 to 0.522) | 0.642 |
| Experience of a genetic condition | -0.727 (-1.343 to -0.111) | 0.021 | 0.032 (-0.444 to 0.509) | 0.894 | 0.382 (-0.056 to 0.82) | 0.088 |
| Experience of AF | 0.145 (-0.513 to 0.803) | 0.666 | -0.067 (-0.592 to 0.459) | 0.803 | -0.054 (-0.547 to 0.438) | 0.829 |
| Experience of other heart conditions | -0.293 (-0.794 to 0.208) | 0.252 | -0.274 (-0.698 to 0.149) | 0.204 | 0.415 (0.015 to 0.816) | 0.042 |
| Knowledge of genetics (Very well, Fair amount) | -0.183 (-0.905 to 0.538) | 0.618 | -0.344 (-0.876 to 0.187) | 0.204 | 0.329 (-0.163 to 0.82) | 0.19 |
| Health score (0-10) | -0.155 (-0.301 to -0.009) | 0.038 | 0.033 (-0.083 to 0.15) | 0.572 | 0.073 (-0.037 to 0.184) | 0.195 |
| Health risk attitudes (0-10) | -0.059 (-0.164 to 0.046) | 0.274 | -0.081 (-0.163 to 0) | 0.05 | 0.106 (0.028 to 0.184) | 0.008 |

Fractional logistic regression analysis to investigate the association between individual characteristics and probability of class membership estimated from Table S3.

**Table S5**. Latent Class Regressions for At Risk Relatives Survey

|  | At risk (n=506) | | | | | |
| --- | --- | --- | --- | --- | --- | --- |
|  | Class 1 (25%, n ≈ 126) | P value | Class 2 (27%, n≈136) | P value | Class 3 (48%, n ≈ 244) | P value |
| Number of people who receive a genetic diagnosis | 0.019 (0.003 to 0.035) | 0.0172 | 0.027 (0.016 to 0.037) | <0.00001 | 0.003 (-0.001 to 0.007) | 0.1086 |
| Knowledge about future recurrence of AF and disease progression (Yes) | 0.272 (-0.099 to 0.642) | 0.1508 | 0.455 (0.247 to 0.663) | <0.00001 | 0.211 (0.128 to 0.294) | <0.00001 |
| Consequences of the genetic diagnosis to you | | | | | | |
| Inform lifestyle changes to avoid triggers of AF | 0.213 (-0.376 to 0.802) | 0.478 | 0.56 (0.203 to 0.917) | 0.0021 | 0.366 (0.204 to 0.528) | <0.00001 |
| Inform changes in treatment of co-morbidities and other risk factors that predisposed to AF | 0.632 (0.093 to 1.171) | 0.0216 | 0.574 (0.252 to 0.896) | 0.0005 | 0.53 (0.374 to 0.686) | <0.00001 |
| Inform ongoing monitoring to detect other heart complications that are related to the genetic diagnosis of AF | 0.557 (-0.059 to 1.174) | 0.0765 | 1.076 (0.66 to 1.492) | <0.00001 | 0.454 (0.285 to 0.622) | <0.00001 |
| Inform changes in AF treatment and management | -0.231 (-0.97 to 0.509) | 0.5408 | 0.707 (0.275 to 1.139) | <0.00001 | 0.338 (0.153 to 0.523) | 0.0003 |
| No consequence | Base | | | | | |
| Consequences of the genetic diagnosis to your family | | | | | | |
| Identify the risk of developing AF and initiate baseline cardiac investigation | 0.696 (0.311 to 1.082) | 0.0004 | 1.013 (0.784 to 1.241) | 0.0013 | 0.452 (0.364 to 0.541) | <0.00001 |
| No consequence | Base | | | | | |
| Cost of testing to you (AU$`1000s) | -0.58 (-0.76 to -0.41) | <0.00001 | -2.29 (-2.58 to -1.99) | <0.00001 | -0.23 (-0.28 to -0.18) | <0.00001 |
| Genomic testing constant | -3.115 (-3.855 to -2.376) | <0.00001 | 1.195 (0.805 to 1.585) | <0.00001 | 2.277 (2.005 to 2.548) | <0.00001 |

Latent class regression analysis to split individuals into a finite number of groups with distinct preferences for genomic testing in AF.

**Table S6**. Fractional Logit Regression for At Risk Relatives Survey

| Individual characteristics | At risk (n=506) | | | | | |
| --- | --- | --- | --- | --- | --- | --- |
|  | Class 1 (25%, n ≈ 126) | P value | Class 2 (27%, n≈136) | P value | Class 3 (48%, n ≈ 244) | P value |
| Aged 45+ years | 0.148 (-0.338 to 0.635) | 0.549 | 0.25 (-0.17 to 0.669) | 0.243 | -0.318 (-0.707 to 0.072) | 0.11 |
| Gender (Female or non-binary) | 0.044 (-0.387 to 0.476) | 0.84 | 0.152 (-0.239 to 0.543) | 0.447 | -0.159 (-0.525 to 0.207) | 0.394 |
| Marital status (Married or in a de facto relationship) | -0.251 (-0.721 to 0.219) | 0.295 | 0.12 (-0.319 to 0.559) | 0.592 | 0.098 (-0.307 to 0.504) | 0.634 |
| Educational status ( Higher education) | -0.149 (-0.652 to 0.354) | 0.561 | 0.374 (-0.047 to 0.795) | 0.082 | -0.223 (-0.63 to 0.185) | 0.284 |
| Annual household income (Over AU$120,000) | -0.005 (-0.516 to 0.505) | 0.984 | -0.151 (-0.592 to 0.29) | 0.503 | 0.125 (-0.284 to 0.535) | 0.549 |
| Children (Yes) | -0.077 (-0.574 to 0.421) | 0.762 | -0.216 (-0.667 to 0.235) | 0.348 | 0.221 (-0.189 to 0.631) | 0.291 |
| Private health insurance (Yes) | -0.537 (-1.008 to -0.066) | 0.026 | 0.519 (0.057 to 0.982) | 0.028 | -0.014 (-0.438 to 0.41) | 0.949 |
| Metropolitan (Yes) | 0.111 (-0.369 to 0.591) | 0.65 | -0.262 (-0.692 to 0.168) | 0.232 | 0.135 (-0.275 to 0.545) | 0.519 |
| Experience of a genetic condition | -0.165 (-0.627 to 0.296) | 0.482 | -0.257 (-0.679 to 0.165) | 0.233 | 0.333 (-0.055 to 0.72) | 0.093 |
| Experience of AF | -0.461 (-1.092 to 0.171) | 0.153 | 0.533 (0.022 to 1.043) | 0.041 | -0.151 (-0.647 to 0.345) | 0.552 |
| Experience of other heart conditions | -0.13 (-0.553 to 0.293) | 0.547 | 0.007 (-0.411 to 0.426) | 0.973 | 0.088 (-0.293 to 0.47) | 0.65 |
| Knowledge of genetics (Very well, Fair amount) | 0.058 (-0.55 to 0.667) | 0.851 | -1.002 (-1.578 to -0.426) | 0.001 | 0.718 (0.22 to 1.216) | 0.005 |
| Health score (0-10) | -0.081 (-0.219 to 0.057) | 0.252 | 0.033 (-0.089 to 0.155) | 0.596 | 0.031 (-0.086 to 0.147) | 0.606 |
| Health risk attitudes (0-10) | 0.01 (-0.081 to 0.101) | 0.831 | -0.072 (-0.153 to 0.009) | 0.082 | 0.055 (-0.024 to 0.134) | 0.173 |

Fractional logistic regression analysis to investigate the association between individual characteristics and probability of class membership estimated from Table S5.


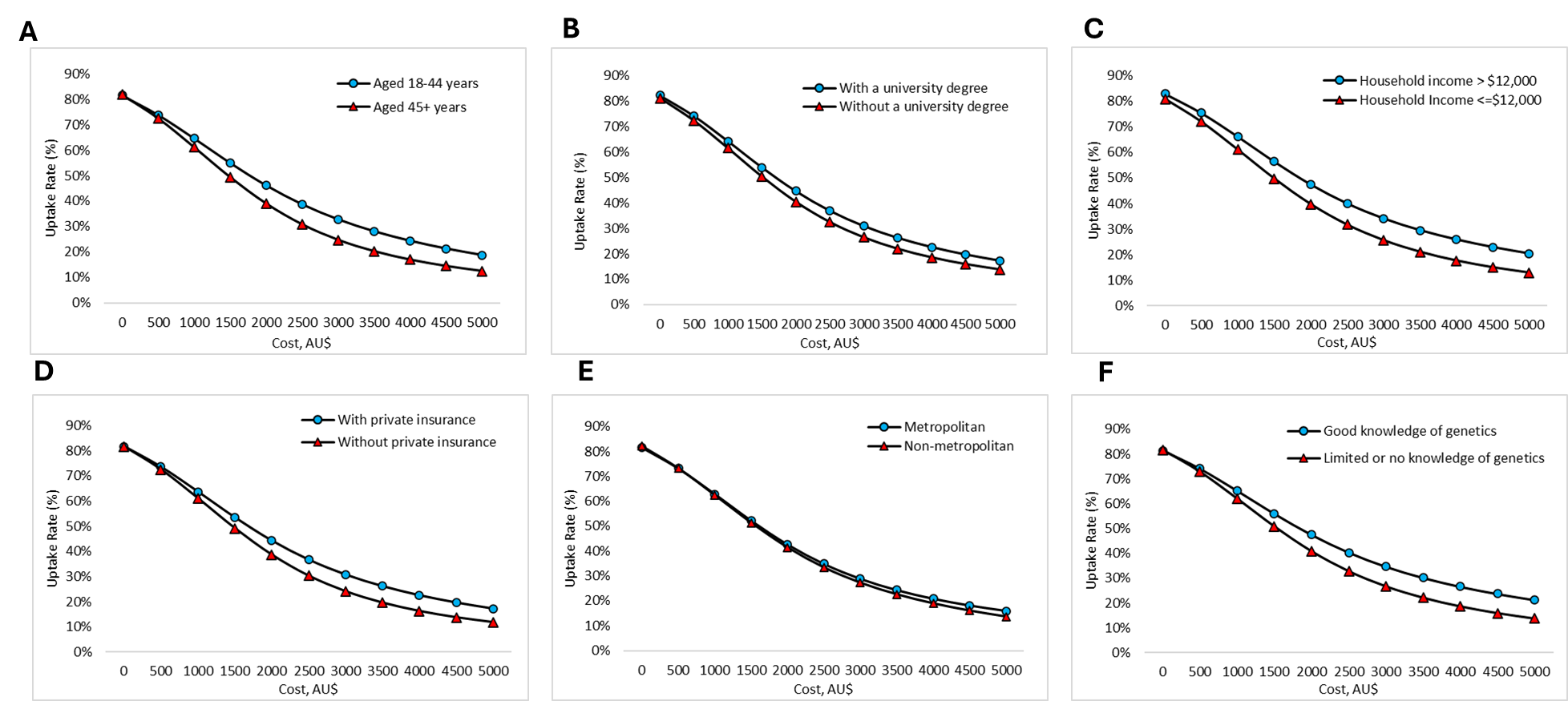


**Figure S1. Uptake rate of genomic testing for AF across different out-of-pocket cost levels in Symptomatic patients survey, by sociodemographic subgroups.** **(A)** uptake rates for individuals aged 18-44 years vs > 45 years; **(B)** uptake rates for individuals with a university degree vs without a university degree; **(C)** uptake rates for individuals with household income > AU$12,000 vs ≤ AU$12,000; **(D)** uptake rates for individuals with a private health insurance vs without a private health insurance; **(E)** uptake rates for individuals residing in metropolitan areas vs non-metropolitan areas; **(F)** uptake rates for individuals with good knowledge of genetics (very well and fair amount) vs limited or no knowledge of genetics (know a little, heard of it, or never heard of it). Assumptions for genomic testing: additional 30 out of 100 individuals could receive a genetic diagnosis for their AF, gene-specific therapy and implications for ablation strategies would be available to 60% of those with a genetic diagnosis, 50% would gain knowledge about disease progression, and family members of those receiving a diagnosis would be advised to be aware of their AF risks


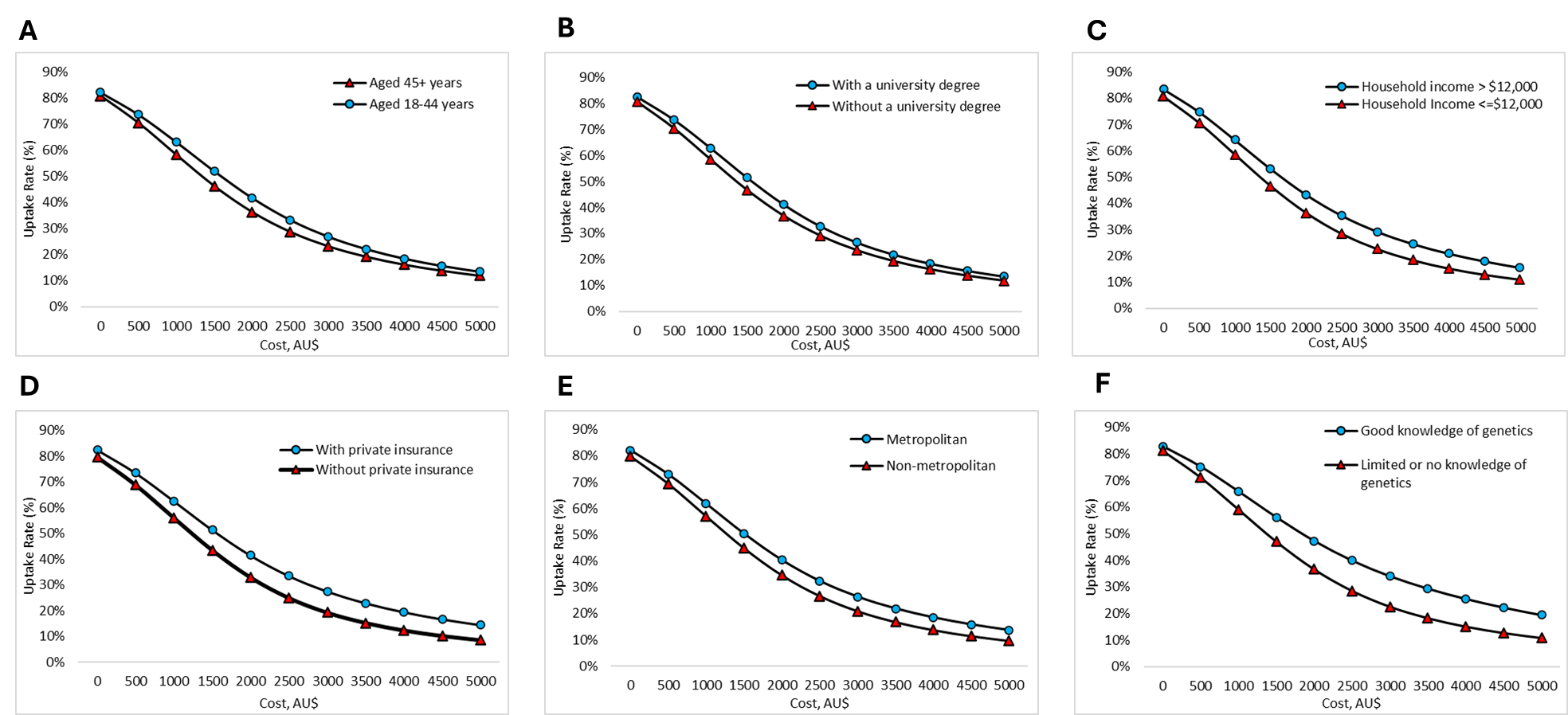


**Figure S2. Uptake rate of genomic testing for AF across different out-of-pocket cost levels in At Risk relatives survey, by sociodemographic subgroups.** **(A)** uptake rates for individuals aged 18-44 years vs > 45 years; **(B)** uptake rates for individuals with a university degree vs without a university degree; **(C)** uptake rates for individuals with household income > AU$12,000 vs ≤ AU$12,000; **(D)** uptake rates for individuals with a private health insurance vs without a private health insurance; **(E)** uptake rates for individuals residing in metropolitan areas vs non-metropolitan areas; **(F)** uptake rates for individuals with good knowledge of genetics (very well and fair amount) vs limited or no knowledge of genetics (know a little, heard of it, or never heard of it). Assumptions for genomic testing: additional 30 out of 100 individuals could receive a genetic diagnosis for their AF, gene-specific therapy and implications for ablation strategies would be available to 60% of those with a genetic diagnosis, 50% would gain knowledge about disease progression, and family members of those receiving a diagnosis would be advised to be aware of their AF risks


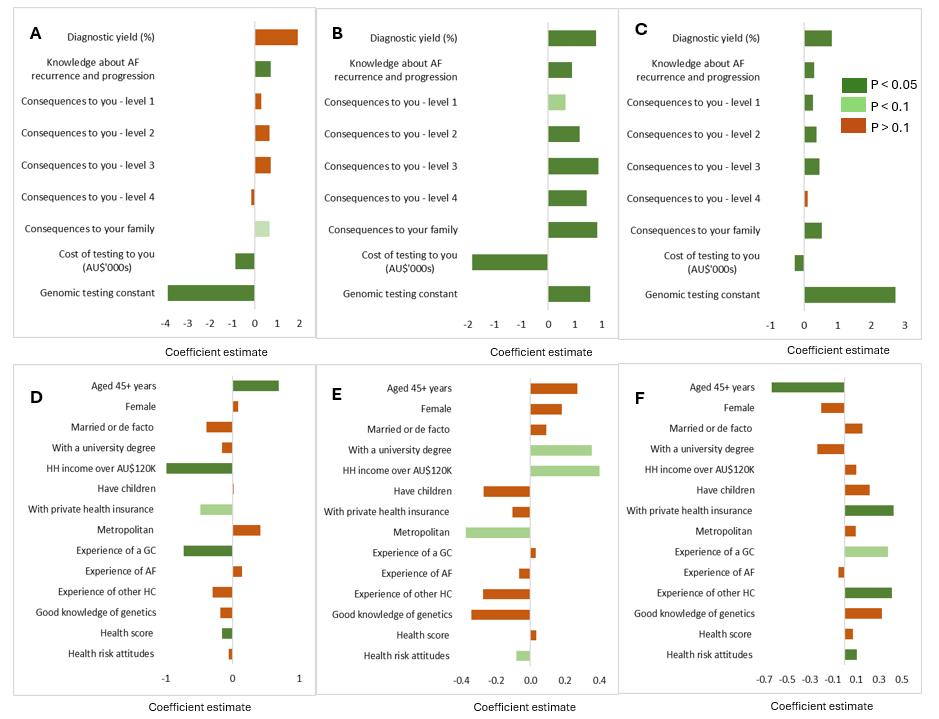


| Class 1 (21%, n ≈ 105) | Class 2 (29%, n≈145) | Class 3 (50%, n ≈ 250) |
| --- | --- | --- |

**Figure S3. Latent class and fractional logit regression for Symptomatic patients survey.** Latent class regression estimates for Class 1 (**A**), Class 2 (**B**) and Class 3 (**C**). Fractional logit regression estimates for Class 1 (**D**), Class 2 (**E**) and Class 3 (**F**). Diagnostic yield (%) indicates percentage of people who receive a genetic diagnosis; Consequences to you level 1 indicates attribute level “Inform changes in AF treatment and management”; Consequences to you level 2 indicates attribute level “Inform ongoing monitoring to detect other heart complications that are related to the genetic diagnosis of AF”; Consequences to you level 3 indicates attribute level “Inform changes in treatment of co-morbidities and other risk factors that predisposed to AF”; Consequences to you level 4 indicates attribute level “Inform lifestyle changes to avoid triggers of AF”; Consequences to your family indicates attribute level “Identify the risk of developing AF and initiate baseline cardiac investigation”. HH income indicates annual household income; and GC, genetic condition; HC heart condition.


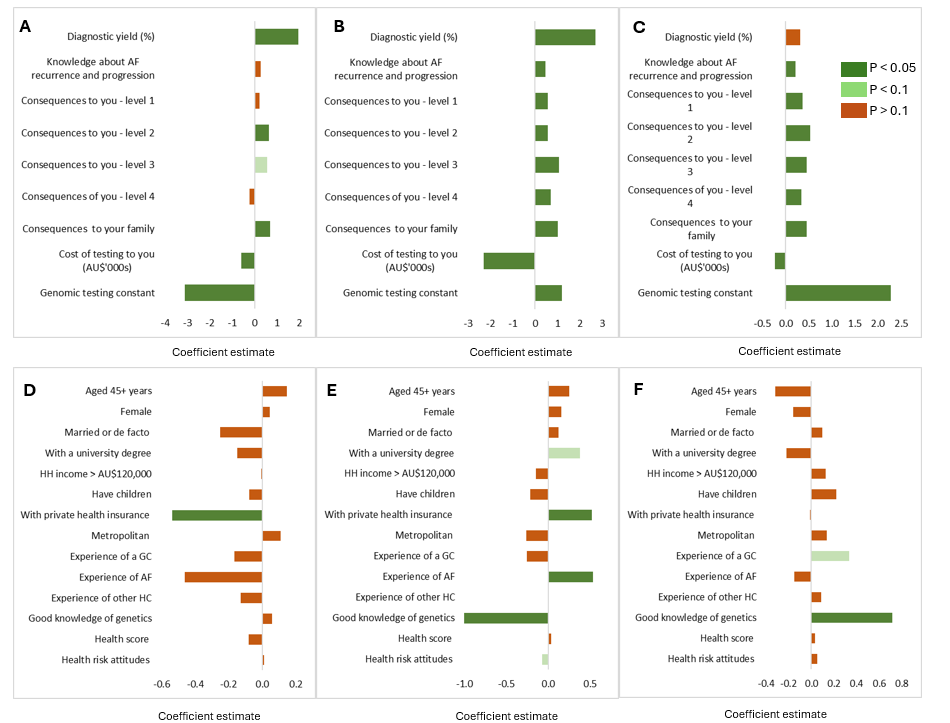


| Class 1 (25%, n ≈ 126) | Class 2 (27%, n≈136) | Class 3 (48%, n ≈ 244) |
| --- | --- | --- |

**Figure S4. Latent class and fractional logit regression for At Risk relatives survey.** Latent class regression estimates for Class 1 (**A**), Class 2 (**B**) and Class 3 (**C**). Fractional logit regression estimates for Class 1 (**D**), Class 2 (**E**) and Class 3 (**F**). Diagnostic yield (%) indicates percentage of people who receive a genetic diagnosis; Consequences to you level 1 indicates attribute level “Inform changes in AF treatment and management”; Consequences to you level 2 indicates attribute level “Inform ongoing monitoring to detect other heart complications that are related to the genetic diagnosis of AF”; Consequences to you level 3 indicates attribute level “Inform changes in treatment of co-morbidities and other risk factors that predisposed to AF”; Consequences to you level 4 indicates attribute level “Inform lifestyle changes to avoid triggers of AF”; Consequences to your family indicates attribute level “Identify the risk of developing AF and initiate baseline cardiac investigation”. HH income indicates annual household income; and GC, genetic condition; HC heart condition
